# Clinical Predictors of Postoperative Medical Complications After Major Abdominal Surgery: A Multicenter Hospital-Based Observational Study

**DOI:** 10.7759/cureus.111588

**Published:** 2026-06-26

**Authors:** Javaria Salam, Kinza Naeem, Ahsan Iqbal, Qurat Ul Ain Malik, Zain Iftikhar, Muhammad Khurram Shahid, Shanza Ijaz, Aymen Shafiq

**Affiliations:** 1 Internal Medicine, Social Security Hospital, Faisalabad, PAK; 2 Medicine and Surgery, District Headquarters (DHQ) Hospital, Mandi Bahauddin, PAK; 3 General Surgery, Islam Teaching Hospital, Gujranwala, PAK; 4 Anesthesia and Critical Care, Shalamar Institute of Health Sciences, Lahore, PAK; 5 Internal Medicine, Central Park Teaching Hospital/Central Park Medical College, Lahore, PAK; 6 Internal Medicine, G.S. Poly Clinic and Al-Nasar Lab Collection Center, Lahore, PAK; 7 Pediatric Anesthesia, Children Hospital, University of Child Health Sciences, Lahore, PAK; 8 Anesthesia, District Headquarters (DHQ) Hospital, Hafizabad, PAK; 9 Surgery, Gujranwala Teaching Hospital, Gujranwala, PAK

**Keywords:** abdominal surgery, clinical predictors, major surgery, multicenter study, observational study, perioperative care, postoperative morbidity, prospective cohort, risk factors, surgical outcomes

## Abstract

Background and aim: Major abdominal surgery is associated with substantial postoperative morbidity, including pulmonary, renal, cardiovascular, and infectious complications. Identification of perioperative factors associated with postoperative medical complications may improve risk stratification and perioperative optimization. The present study aimed to identify predictors of postoperative medical complications in patients undergoing major abdominal surgery, with the goal of improving perioperative risk stratification and informing strategies to reduce postoperative morbidity.

Methods: This multicenter prospective observational cohort study was conducted at two tertiary care hospitals in Lahore, Pakistan. Adult patients undergoing major abdominal surgery were enrolled consecutively over a 12-month period. The primary outcome was the occurrence of postoperative medical complications within 30 days of surgery. Univariate and multivariate logistic regression analyses were performed to identify independent predictors of postoperative medical complications.

Results: A total of 284 patients were included, of whom 96 (33.8%) developed postoperative medical complications. Pulmonary complications were the most common events, followed by acute kidney injury and systemic infection. Patients with complications had significantly longer hospital stays (11 vs. six days, p<0.001). On multivariable analysis, American Society of Anesthesiologists (ASA) ≥III, hypoalbuminemia, emergency surgery, prolonged operative duration, intraoperative hypotension, and blood transfusion were independently associated with postoperative medical complications.

Conclusions: Postoperative medical complications remain common after major abdominal surgery. Both patient-related and perioperative factors were independently associated with postoperative morbidity. Early identification of high-risk patients and optimization of modifiable perioperative factors may help reduce postoperative medical complications and improve surgical outcomes.

## Introduction

Major abdominal surgery remains a cornerstone of treatment for many gastrointestinal, hepatobiliary, and pancreatic diseases, yet it is consistently associated with substantial postoperative morbidity [1]. In addition to technical surgical complications, patients frequently develop postoperative medical complications involving major organ systems, including pulmonary complications, acute kidney injury (AKI), myocardial injury, delirium, venous thromboembolism, and sepsis [2]. These complications significantly prolong hospital stay, delay recovery, and increase mortality risk, even when overall operative mortality remains relatively low. In a large cohort of patients undergoing major abdominal procedures, such as colorectal, hepatic, small bowel resections, and pancreaticoduodenectomy, more than 75% experienced at least one postoperative complication, with 15.5% developing major complications, with healthcare costs increasing steeply as the number and severity of complications rose [3].

The occurrence of postoperative medical complications reflects a complex interaction between patient vulnerability, procedural stress, and perioperative care quality. Several patient-related factors have been identified as important predictors, including advanced age, comorbid disease burden, sarcopenia, and malnutrition [4,5]. Reduced physiological reserve in these populations increases susceptibility to postoperative organ dysfunction and impaired recovery. For example, sarcopenia has been associated with increased postoperative complications and mortality after abdominal surgery [5], while malnutrition and hypoalbuminemia significantly increase postoperative morbidity risk [6]. In addition to baseline patient factors, intraoperative exposures also play a critical role. Longer operative duration, open surgical approaches, blood loss, transfusion requirements, and intraoperative hypotension have been linked to higher rates of complications [7]. In particular, greater exposure to intraoperative hypotension has been associated with increased risk of postoperative AKI, while perioperative red blood cell transfusion has been linked with higher postoperative morbidity and infectious complications [8].

Perioperative management strategies also influence the development of postoperative medical complications. Early postoperative pain and inadequate analgesia may impair respiratory mechanics and contribute to pulmonary complications, whereas effective analgesic strategies such as patient-controlled analgesia have been associated with reduced pulmonary complication rates in elderly patients undergoing major upper abdominal surgery [7,9]. Similarly, optimization of hemodynamic stability, avoidance of nephrotoxic medications, and adherence to enhanced recovery pathways are increasingly recognized as important modifiable factors in reducing postoperative morbidity. Despite these advances, the incidence of organ-system complications remains considerable. For example, postoperative pulmonary complications occur in approximately 12% of high-risk abdominal surgeries [10], while a recent meta-analysis reported an overall AKI incidence of approximately 16% following abdominal procedures, both of which are strongly associated with increased short- and long-term mortality [11].

Several risk prediction tools have been developed to identify patients at increased risk of adverse postoperative outcomes, including widely used models such as Physiological and Operative Severity Score for the enUmeration of Mortality and morbidity (POSSUM) and the Surgical Outcome Risk Tool (SORT) [12,13]. Although these models demonstrate reasonable discrimination in some settings, their performance varies significantly across different populations and healthcare systems. External validation studies have shown that some models may underestimate risk in high-risk groups, while systematic reviews of prediction models for postoperative pulmonary complications have highlighted substantial heterogeneity, limited external validation, and frequent methodological limitations [13]. Furthermore, many existing models focus on composite surgical outcomes rather than specifically addressing postoperative medical complications, and important predictors such as frailty, sarcopenia, and nutritional status are often inconsistently measured or omitted.

Given the substantial burden of postoperative medical complications and the limitations of existing prediction models, further research is needed to better identify clinically relevant predictors in contemporary surgical populations. Understanding these predictors may allow earlier identification of high-risk patients and support targeted perioperative optimization strategies.

## Materials and methods

Study design and setting

This multicenter prospective observational cohort study, aimed at identifying predictors of postoperative medical complications in patients undergoing major abdominal surgery, was conducted from April 2025 to April 2026 after receiving ethical approval from the Institutional Review Board of Gujranwala Medical College (IRB/21/GMC) in April 2025. Patient recruitment and data collection were performed at two tertiary care hospitals in Gujranwala and Lahore, Pakistan, both of which have high surgical volumes and were covered by the aforementioned IRB. Consecutive eligible patients were enrolled at both centers over a 12-month study period. Written informed consent was obtained from all patients prior to inclusion in the study. The study was conducted in accordance with the principles of the Declaration of Helsinki [14]. Investigators from additional collaborating institutions contributed to study design, protocol development, data monitoring, and statistical analysis, while patient recruitment remained limited to the two primary centers to maintain uniformity in data collection and ethical oversight.

Sample size calculation

The sample size was determined based on the expected number of postoperative medical complication events required for multivariable logistic regression analysis. As this was an observational study designed to identify predictors rather than test an intervention, the sample size was guided by the events-per-variable principle. A minimum of 10 outcome events per predictor variable was considered acceptable for logistic regression modeling. Based on previously reported postoperative complication rates after major abdominal surgery of approximately 30%, a sample size of at least 270-280 patients was considered sufficient to yield approximately 80-85 complication events, allowing reliable assessment of eight to 10 clinically relevant predictor variables in the multivariable model. During the 12-month recruitment period, 312 patients were assessed, and 284 patients were included in the final analysis after exclusions.

Study population

Adult patients undergoing major abdominal surgery were considered eligible for inclusion. For this study, major abdominal surgery was defined as a therapeutic intra-abdominal operation performed under general anesthesia that involved intraperitoneal access and was expected to require postoperative inpatient care for more than 48 h.

Eligible procedures included colorectal resections, small bowel resections, stricturoplasty, adhesiolysis for bowel obstruction, stoma creation or reversal, repair of gastrointestinal perforation, emergency laparotomy for peritonitis or intra-abdominal sepsis, major gastric procedures, hepatobiliary surgery, pancreatic surgery, and other therapeutic gastrointestinal operations involving intraperitoneal access. Both elective and emergency surgeries were included.

Patients were excluded if they underwent minor or purely diagnostic abdominal procedures, such as diagnostic laparoscopy without therapeutic intervention, or if they were discharged within 24 h following surgery. Patients undergoing primarily vascular, urological, abdominal wall, or extra-peritoneal procedures without intraperitoneal access were excluded. Patients with incomplete perioperative data or those who declined participation were also excluded from the analysis.

Outcome measures

The primary outcome of interest was the occurrence of postoperative medical complications within 30 days following surgery. Medical complications were defined as non-technical, organ-system complications occurring after the index operation. To reduce ascertainment bias, predefined operational criteria were used for each component of the composite outcome.

Pulmonary complications included postoperative pneumonia, respiratory failure requiring ventilatory support, or re-intubation. Postoperative pneumonia was defined as new or progressive pulmonary infiltrates on chest imaging together with compatible clinical features, including fever, leukocytosis or leukopenia, purulent respiratory secretions, worsening oxygen requirement, or initiation of antibiotic therapy for suspected lower respiratory tract infection. Respiratory failure was defined as postoperative respiratory compromise requiring non-invasive ventilation, invasive mechanical ventilation, or re-intubation after initial extubation.

Acute kidney injury was defined according to the Kidney Disease: Improving Global Outcomes criteria, based on a postoperative increase in serum creatinine and/or a reduction in urine output, when documented. Myocardial injury was defined as postoperative elevation of cardiac biomarkers above the institutional upper reference limit. Myocardial infarction required biomarker elevation with additional evidence of myocardial ischemia, such as ischemic symptoms, electrocardiographic changes, imaging evidence, or cardiology documentation.

Delirium was defined as an acute postoperative disturbance of attention, awareness, or cognition, as documented in the medical record. Formal delirium screening tools such as the Confusion Assessment Method (CAM), CAM-ICU, or 4 ‘A’s Test (4AT) were not routinely applied across all sites; therefore, delirium was recorded only when documented by the treating team as an acute confusional state, delirium, agitation with altered attention, or fluctuating cognitive disturbance.

Venous thromboembolism was defined as deep vein thrombosis or pulmonary embolism confirmed by radiological imaging. Systemic infection was defined as suspected or confirmed infection requiring antimicrobial therapy together with systemic clinical or laboratory evidence of infection, such as fever, leukocytosis or leukopenia, raised inflammatory markers, positive cultures, or imaging evidence of infection. Sepsis was defined in line with the Sepsis-3 principles as a suspected or confirmed infection associated with acute organ dysfunction, hemodynamic instability, the requirement for vasopressor support, ICU escalation, or other evidence of infection-related clinical deterioration.

Postoperative complications were graded using the Clavien-Dindo classification system according to the treatment required to manage the complication. The Clavien-Dindo system was used for severity grading after a complication had been identified and was not used as the diagnostic criterion for sepsis or other medical complications. Clinically significant complications were defined as those corresponding to Clavien-Dindo grade III or higher.

Study variables

Study variables were grouped conceptually into preoperative, intraoperative, and postoperative/perioperative variables. Preoperative variables included demographic and clinical characteristics, American Society of Anesthesiologists (ASA) physical status classification, relevant comorbidities, and baseline laboratory parameters, including hemoglobin and serum albumin. Intraoperative variables included urgency of surgery, surgical approach, operative duration, intraoperative blood transfusion, and intraoperative hypotension. Postoperative/perioperative clinical-course variables included ICU admission and postoperative pain control within the first 24 h.

Only variables with adequate data completeness, clinical relevance, and inclusion in the planned comparative analysis were presented in the subsequent sections of the study. Variables recorded inconsistently across centers or not included in the planned analysis were not entered into the univariate or multivariable models.

Marked hypoalbuminemia was defined as serum albumin <3.0 g/dL. Prolonged operative duration was defined as surgery lasting more than 180 min. Intraoperative hypotension was defined as mean arterial pressure <65 mmHg for at least 2 min or any episode of mean arterial pressure (MAP) <65 mmHg requiring intervention with intravenous fluids, vasopressors, or anesthetic adjustment. Poor postoperative pain control was defined as a numerical rating scale score ≥4 within the first 24 h postoperatively. Blood transfusion refers to any intraoperative administration of packed red blood cells or whole blood.

Data collection

Data were collected prospectively using a standardized, structured data collection proforma developed specifically for the study. Relevant information was obtained from patient medical records, anesthesia charts, operative notes, laboratory reports, and postoperative monitoring records. At each participating center, designated investigators were responsible for data collection and verification to ensure accuracy and completeness. All collected data were anonymized prior to analysis and entered into a secure electronic database accessible only to authorized study investigators.

All enrolled patients were followed for 30 days after surgery to identify postoperative medical complications. During the hospital stay, patients were reviewed regularly, and postoperative events were recorded. For patients discharged before completion of the 30-day follow-up period, outcome data were obtained through review of hospital records and telephone contact when necessary to ensure complete follow-up.

Statistical analysis

Statistical analysis was performed using SPSS version 26.0 (Armonk, NY: IBM Corp.). Continuous variables were assessed for normality using graphical methods and summarized as mean±standard deviation or median with interquartile range, as appropriate. Categorical variables were expressed as frequencies and percentages. Associations between potential predictor variables and the occurrence of postoperative medical complications were initially assessed using univariate analysis. The chi-square test or Fisher’s exact test was used for categorical variables, while the independent t-test or Mann-Whitney U test was applied for continuous variables as appropriate.

Because only two centers were included, random-effects modeling was not performed; instead, center-level comparability was assessed descriptively, and pooled analysis was undertaken after confirming broadly similar baseline characteristics, perioperative variables, and 30-day postoperative complication rates across the two hospitals.

Variables demonstrating a p-value of less than 0.10 in univariate analysis, together with clinically relevant variables, were initially considered as candidate variables for multivariable logistic regression. Variables were interpreted according to their temporal relationship with surgery and the primary outcome. Preoperative and intraoperative factors were considered candidate predictors for the primary multivariable risk model. Postoperative variables, including ICU admission and poor pain control within the first 24 h, were analyzed descriptively and in univariate analysis as clinical-course variables associated with postoperative complications, but were not included in the final multivariable predictor model because of potential reverse causality and temporal ambiguity.

Because 96 patients developed postoperative medical complications, inclusion of all 12 candidate variables would have resulted in approximately eight outcome events per variable, increasing the risk of overfitting. Therefore, a restricted multivariable logistic regression model was constructed using a purposeful selection approach. Variables were selected for the final model based on clinical relevance, temporal relationship to the outcome, strength of univariate association, and avoidance of overfitting. Postoperative variables that could plausibly represent consequences or markers of complication severity, such as ICU admission and poor postoperative pain control, were not included in the primary predictor model.

The final multivariable logistic regression model included six variables. Adjusted odds ratios with 95% confidence intervals were reported. A supplementary candidate-variable table was added to show all variables considered for multivariable analysis, whether they were included in the final model, and the reason for exclusion where applicable. A p<0.05 was considered statistically significant.

Multicollinearity among candidate variables was assessed before construction of the multivariable logistic regression model using variance inflation factor (VIF) and tolerance values. A VIF >5 or tolerance <0.20 was considered suggestive of potentially problematic multicollinearity. Variables showing substantial collinearity were reviewed clinically before inclusion in the final model.

## Results

Baseline characteristics

**Figure 1:**
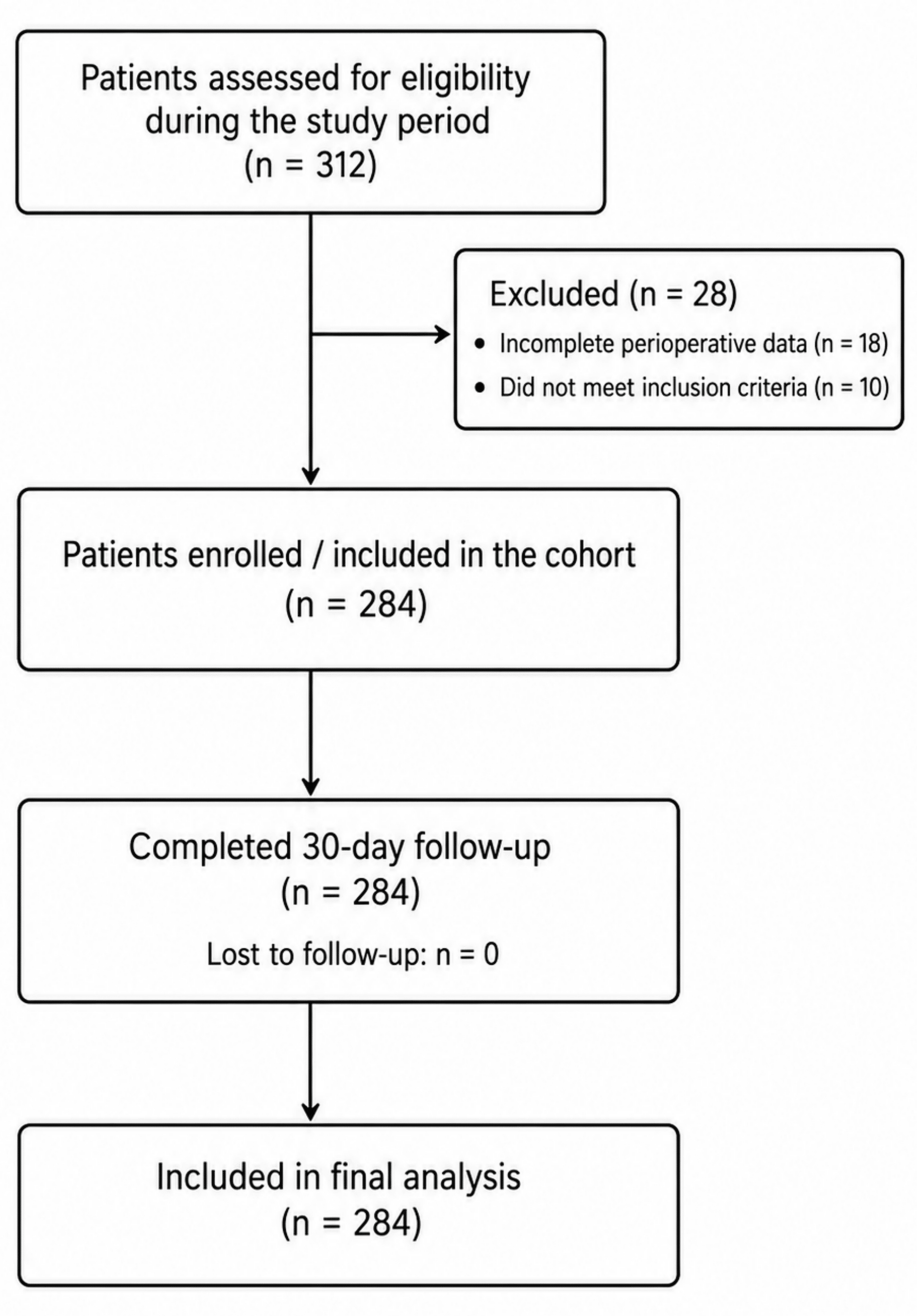
Patient flow diagram (prepared in accordance with STROBE reporting recommendations). STROBE: Strengthening the Reporting of Observational Studies in Epidemiology

Figure 1 shows the patient flow diagram, which includes screening, eligibility assessment, follow-up, and final inclusion of patients in the analysis. A total of 312 patients undergoing major abdominal surgery were assessed for eligibility during the study period. A total of 28 patients were excluded due to incomplete perioperative data (n=18) or failure to meet inclusion criteria (n=10). Consequently, 284 patients were included in the final analysis. Of the included patients, approximately equal numbers were recruited from both participating centers.

Patients were recruited from two tertiary care hospitals. Approximately equal numbers of patients were recruited from both participating centers. Both centers used the same eligibility criteria, data collection proforma, operational outcome definitions, and 30-day follow-up procedure. Center-level baseline characteristics and postoperative complication rates were reviewed descriptively and were found to be broadly comparable between the two hospitals. Therefore, pooled analysis was considered appropriate. Complete 30-day follow-up data were available for all included patients.

The analyzed variables are presented according to their temporal relationship with surgery. Preoperative factors included demographic characteristics, comorbidities, ASA classification, hemoglobin, and serum albumin. Intraoperative factors included urgency of surgery, operative approach, operative duration, blood transfusion, and intraoperative hypotension. Postoperative/perioperative clinical-course variables included ICU admission and pain control within the first 24 h. The baseline characteristics of the study population are summarized in Table 1.

**Table 1:** Baseline characteristics of the study population and comparison between patients with and without postoperative medical complications. ASA: American Society of Anesthesiologists

Variables	Overall (n=284)	No complications (n=188)	Complications (n=96)	p-Value
Age (years), mean±SD	52.6±14.8	49.8±13.9	57.9±15.2	0.001
Age >60 years, n (%)	102 (35.9)	55 (29.3)	47 (49.0)	0.002
Male sex, n (%)	168 (59.2)	114 (60.6)	54 (56.3)	0.49
BMI (kg/m²), mean±SD	26.1±4.2	25.8±4.0	26.6±4.5	0.18
ASA ≥III, n (%)	108 (38.0)	52 (27.7)	56 (58.3)	<0.001
Diabetes mellitus, n (%)	88 (31.0)	50 (26.6)	38 (39.6)	0.028
Hypertension, n (%)	104 (36.6)	62 (33.0)	42 (43.8)	0.08
Ischemic heart disease, n (%)	36 (12.7)	18 (9.6)	18 (18.8)	0.031
Hypoalbuminemia, n (%)	78 (27.5)	32 (17.0)	46 (47.9)	<0.001
Hemoglobin (g/dL), mean±SD	11.4±1.8	11.6±1.7	11.1±1.9	0.07
Emergency surgery, n (%)	106 (37.3)	58 (30.9)	48 (50.0)	0.004
Open surgery, n (%)	174 (61.3)	108 (57.4)	66 (68.8)	0.064
Duration >180 min, n (%)	92 (32.4)	48 (25.5)	44 (45.8)	0.001
Blood transfusion, n (%)	67 (23.6)	30 (16.0)	37 (38.5)	<0.001
Intraoperative hypotension, n (%)	81 (28.5)	41 (21.8)	40 (41.7)	0.002

The mean age of the cohort was 52.6±14.8 years, with a predominance of male patients (n=168 {59.2%}). A significant proportion of patients were elderly, with 35.9% aged over 60 years. A substantial proportion of patients were classified as ASA ≥III (n=108 {38.0%}), reflecting a cohort with moderate to high perioperative risk.

Comorbidities were common, with 88 patients (31.0%) having diabetes mellitus, 104 (36.6%) having hypertension, and 36 (12.7%) having ischemic heart disease. Nutritional assessment showed that 78 patients (27.5%) had marked hypoalbuminemia (<3.0 g/dL), indicating a high prevalence of preoperative hypoalbuminemia in the study population. Regarding operative characteristics, 106 procedures (37.3%) were performed on an emergency basis, while 174 (61.3%) were carried out using an open surgical approach. The mean operative duration was 168±52 min, with 92 (32.4%) procedures exceeding 180 min.

Incidence and pattern of postoperative medical complications

Within 30 days following surgery, 96 (33.8%) patients developed at least one postoperative medical complication. Patients with complications experienced a significantly longer median hospital stay compared to those without complications (11 days {IQR: 8-16} vs. six days {IQR 4-9}, p<0.001).

**Figure 2:**
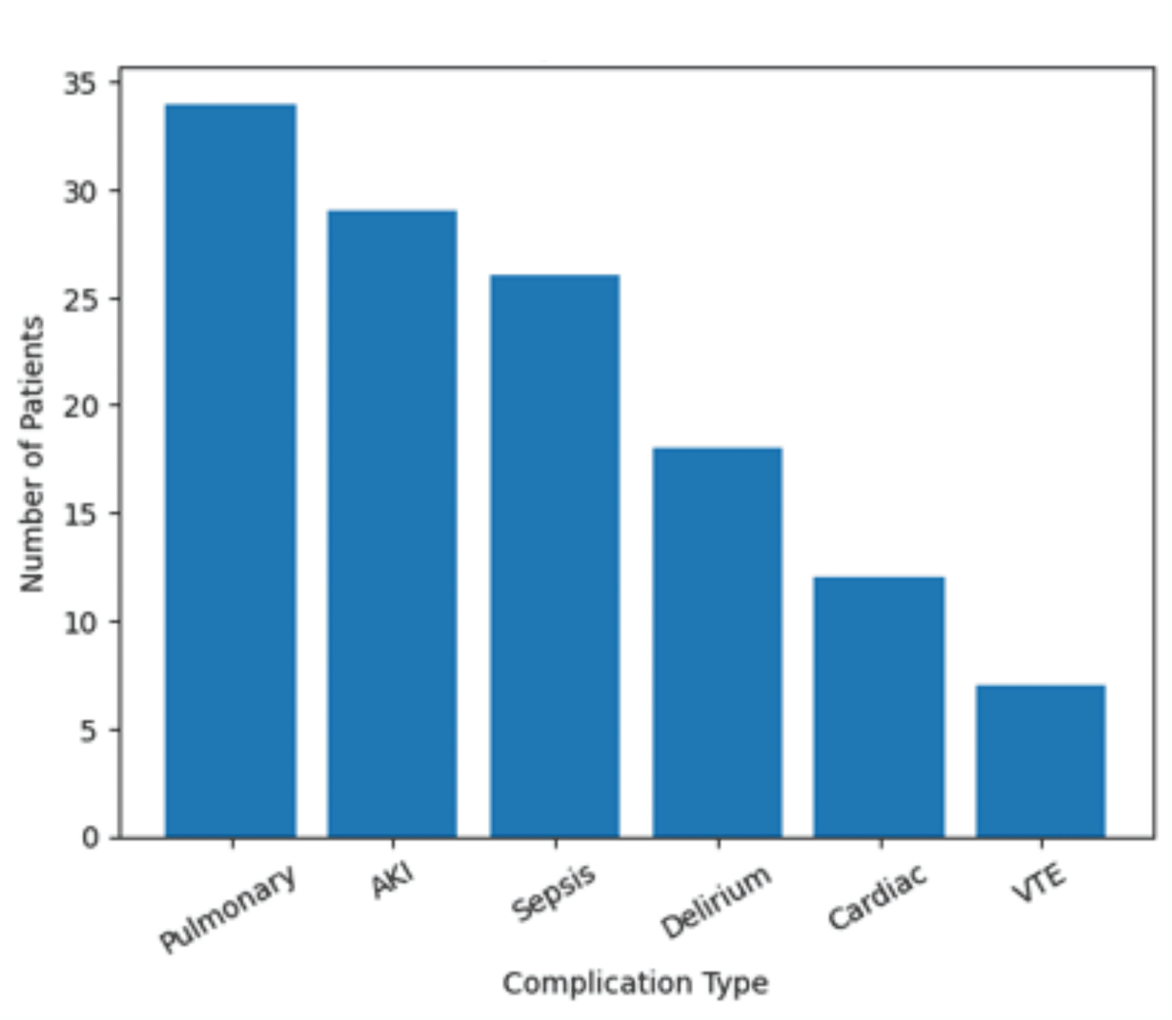
Distribution of postoperative medical complications. Pulmonary complications were the most commonly observed postoperative medical complications, followed by acute kidney injury and systemic infection. Complication categories were not mutually exclusive, as some patients experienced more than one postoperative medical complication. AKI: acute kidney injury; VTE: venous thromboembolism

Pulmonary complications were the most frequently observed events, followed by acute kidney injury and systemic infection. Less common complications included delirium, cardiac events, and venous thromboembolism. Among patients who developed complications, 64 (66.7%) experienced clinically significant complications corresponding to Clavien-Dindo grade III or higher. The distribution of postoperative medical complications is illustrated in Figure 2.

Comparison between patients with and without complications

A comparison of baseline and perioperative characteristics between patients with and without postoperative complications is presented in Table 1. Patients who developed complications were significantly older than those without complications (57.9±15.2 vs. 49.8±13.9 years, p=0.001) and were more frequently classified as ASA ≥III (n=56, 58.3% vs. n=52, 27.7%; p<0.001).

Marked hypoalbuminemia was significantly more prevalent among patients with complications compared with those without complications (n=46, 47.9% vs. n=32, 17.0%; p<0.001). Emergency surgery was also significantly more common in the complication group (n=48, 50.0% vs. n=58, 30.9%; p=0.004). Intraoperative factors, including blood transfusion (n=37, 38.5% vs. n=30, 16.0%; p<0.001) and intraoperative hypotension (n=40, 41.7% vs. n=41, 21.8%; p=0.002), were more frequently observed in patients who developed complications. There were no statistically significant differences between groups in terms of sex distribution or body mass index.

Regression Analysis

Univariate logistic regression analysis identified several factors associated with an increased risk of postoperative medical complications (Table 2). Strong associations were observed for marked hypoalbuminemia, higher ASA classification, intraoperative blood transfusion, and ICU admission. Additional factors, including advanced age, emergency surgery, prolonged operative duration, and intraoperative hypotension, were significantly associated with postoperative complications. The open surgical approach showed a trend toward association and was considered for multivariable analysis because its p-value was below 0.10. Variables demonstrating a p-value of less than 0.10 were considered for inclusion in the multivariable logistic regression model. The detailed multivariable regression model, including the variables entered into the analysis is provided in table in appendix.

**Table 2:** Univariate logistic regression analysis of factors associated with postoperative medical complications. ICU admission and poor pain control within the first 24 h were postoperative clinical-course variables. They were included in univariate comparisons for descriptive purposes but were not included in the final multivariable predictor model because of potential reverse causality and temporal ambiguity.

Variables	Odds ratio	95% CI	p-Value
Age >60 years	2.31	1.36-3.93	0.002
ASA ≥III	3.64	2.13-6.21	<0.001
Diabetes mellitus	1.81	1.07-3.06	0.028
Ischemic heart disease	2.17	1.07-4.40	0.031
Hypoalbuminemia	4.43	2.57-7.64	<0.001
Emergency surgery	2.23	1.33-3.73	0.004
Open surgery	1.63	0.97-2.74	0.064
Duration >180 min	2.52	1.48-4.27	0.001
Blood transfusion	3.30	1.87-5.82	<0.001
Intraoperative hypotension	2.56	1.50-4.37	0.001
ICU admission	3.88	2.21-6.80	<0.001
Poor pain control	1.92	1.15-3.20	0.011

Postoperative clinical-course variables, including ICU admission and poor pain control within the first 24 h, were also more frequent among patients who developed postoperative medical complications. However, these variables were interpreted as markers of postoperative course and illness severity rather than independent baseline predictors. ICU admission may reflect escalation of care due to early postoperative deterioration or complications, while poor pain control represents an early postoperative measure. Therefore, these variables were included in univariate comparisons for descriptive purposes but were not included in the final multivariable predictor model because of potential reverse causality and temporal ambiguity.

Variables identified in univariate analysis were considered for inclusion in the multivariable model after assessing for collinearity. Multicollinearity assessment did not demonstrate problematic collinearity among variables included in the final multivariable logistic regression model. No included variable exceeded the predefined variance inflation factor (VIF) threshold of >5, and no tolerance value fell below the predefined threshold of <0.20. These findings supported retention of the selected predictors in the final model. Multivariable logistic regression analysis identified several independent predictors of postoperative medical complications (Table 3).

**Table 3:** Multivariable logistic regression analysis identifying independent predictors of postoperative medical complications.

Variables	Adjusted OR	95% CI	p-Value
ASA ≥III	2.41	1.35-4.29	0.003
Hypoalbuminemia	2.78	1.56-4.95	<0.001
Emergency surgery	1.92	1.08-3.41	0.025
Duration >180 min	2.15	1.22-3.78	0.008
Intraoperative hypotension	2.36	1.31-4.26	0.004
Blood transfusion	2.89	1.52-5.49	0.001

**Figure 3:**
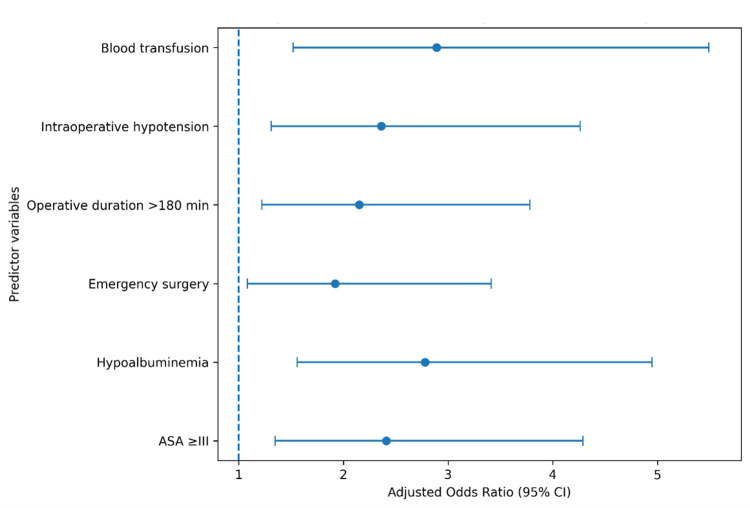
Forest plot of independent predictors of postoperative medical complications. Forest plot demonstrating adjusted odds ratios with 95% confidence intervals for independent predictors of postoperative medical complications identified on multivariable logistic regression analysis. ASA: American Society of Anesthesiologists

Hypoalbuminemia and intraoperative blood transfusion emerged as the strongest independent predictors. Higher ASA classification, intraoperative hypotension, prolonged operative duration, and emergency surgery were also independently associated with increased risk. Age and surgical approach did not retain statistical significance after adjustment for confounding variables. The relative strength of independent predictors identified in the multivariable model is shown in Figure 3.

## Discussion

This multicenter prospective observational study identified several clinically relevant predictors of postoperative medical complications following major abdominal surgery. In this cohort, 33.8% (n=96) of patients developed at least one postoperative medical complication within 30 days. Pulmonary complications were the most frequent events, followed by acute kidney injury and systemic infection. On multivariable analysis, ASA physical status ≥III, hypoalbuminemia, emergency surgery, operative duration greater than 180 min, intraoperative hypotension, and intraoperative blood transfusion were independently associated with postoperative medical complications. These findings suggest that postoperative medical morbidity after major abdominal surgery is influenced by both baseline patient vulnerability and potentially modifiable perioperative factors.

The observed complication rate is consistent with the substantial morbidity reported after major abdominal and gastrointestinal surgery. Jakobson et al. reported postoperative complications in 33.5% of patients undergoing major gastrointestinal surgery, with higher rates among high-risk patients [15]. Similarly, Armellini et al. demonstrated a high burden of complications after major abdominal surgery and showed that complication frequency and severity were associated with increasing healthcare costs [3]. The complication rate in the present study is therefore clinically plausible, particularly because the cohort included both elective and emergency procedures and focused specifically on postoperative medical complications rather than purely technical surgical events. The longer hospital stay observed among patients with complications further supports the clinical relevance of these outcomes and is consistent with the established association between postoperative morbidity and increased resource utilization.

Higher ASA physical status emerged as an independent predictor of postoperative medical complications. This finding is expected, as ASA classification reflects the patient’s overall preoperative physiological reserve and systemic disease burden. Patients with ASA ≥III are more likely to have reduced cardiopulmonary reserve, impaired metabolic response to surgical stress, and limited ability to compensate for perioperative insults. Previous studies have similarly demonstrated the value of ASA status in identifying patients at increased risk of postoperative morbidity and mortality after major abdominal surgery [13,15]. In the present study, the persistence of ASA ≥III as an independent predictor after adjustment suggests that global preoperative risk assessment remains important even when individual comorbidities and intraoperative factors are considered.

Hypoalbuminemia was one of the strongest independent predictors of postoperative medical complications. Serum albumin is commonly used as a marker of nutritional and inflammatory status, and low albumin levels may reflect malnutrition, systemic inflammation, chronic disease burden, or a combination of these factors. Malnutrition and impaired body composition have repeatedly been associated with adverse postoperative outcomes after abdominal surgery. Park et al. reported that consensus-defined sarcopenia predicted adverse outcomes after elective abdominal surgery, while Shen et al. found that Global Leadership Initiative on Malnutrition (GLIM)-defined malnutrition was associated with postoperative complications and worse long-term prognosis in elderly patients undergoing colorectal cancer surgery [5,6]. Although serum albumin should not be interpreted as a pure nutritional marker, its independent association with complications in this study supports its usefulness as a simple and widely available preoperative risk marker. These findings reinforce the importance of nutritional assessment and optimization before major abdominal surgery whenever clinically feasible.

Emergency surgery was also independently associated with postoperative medical complications. Emergency procedures are frequently performed in patients with acute physiological derangement, sepsis, dehydration, bowel obstruction, perforation, or limited time for preoperative optimization. Compared with elective surgery, emergency operations often occur under less controlled conditions and may involve greater hemodynamic instability, contamination, and postoperative organ stress. This finding is clinically important because it highlights the need for structured perioperative pathways for emergency surgical patients, including early resuscitation, timely antibiotics when indicated, careful hemodynamic management, and postoperative monitoring in appropriate care settings. While enhanced recovery pathways have been most strongly established in elective colorectal surgery, the principles of early optimization, goal-directed care, multimodal analgesia, and early mobilization remain relevant to emergency abdominal surgery where feasible [1].

Operative duration greater than 180 min was another independent predictor of postoperative medical complications. Longer operative time may reflect procedural complexity, difficult anatomy, greater tissue trauma, blood loss, fluid shifts, prolonged anesthetic exposure, and increased inflammatory response. Prolonged surgery may also contribute to hypothermia, impaired respiratory mechanics, and delayed postoperative recovery. In this study, the association between longer operative duration and medical complications remained significant after adjustment, suggesting that operative time may act as a practical marker of intraoperative burden. Although operative duration is not always modifiable, anticipation of prolonged surgery should prompt careful perioperative planning, including temperature control, fluid management, blood conservation strategies, postoperative respiratory care, and consideration of higher-acuity postoperative monitoring.

Intraoperative hypotension was independently associated with postoperative medical complications. This finding is biologically plausible because sustained reductions in mean arterial pressure can compromise renal, myocardial, and cerebral perfusion, particularly in patients with limited physiological reserve. Fujii et al. reported that intraoperative hypotension was associated with postoperative acute kidney injury depending on the invasiveness of abdominal surgery [8]. A recent systematic review and meta-analysis also reported an overall acute kidney injury (AKI) incidence of approximately 16% after abdominal surgery and highlighted the importance of perioperative risk factors in the development of postoperative renal injury [11]. In the present study, the association between intraoperative hypotension and postoperative medical complications supports the importance of vigilant hemodynamic monitoring and timely correction of hypotension during major abdominal surgery.

Intraoperative blood transfusion was also an independent predictor of postoperative medical complications. This association should be interpreted carefully, as transfusion may be both a marker of greater surgical complexity or blood loss and a contributor to postoperative morbidity. Nevertheless, existing evidence suggests that perioperative red blood cell transfusion is associated with poorer short- and long-term outcomes after major abdominal surgery. Morris et al. reported that perioperative red blood cell transfusion in elective major abdominal surgery was associated with increased morbidity, infectious complications, and mortality [16]. The findings of the present study, therefore, support the importance of patient blood management strategies, including preoperative correction of anemia when possible, meticulous hemostasis, restrictive transfusion practices where appropriate, and minimization of avoidable blood loss.

Pulmonary complications were the most frequently observed medical complications in this cohort. This is consistent with previous literature showing that postoperative pulmonary complications remain a major source of morbidity after upper and major abdominal surgery. He et al. reported that postoperative pulmonary complications occur in elderly patients undergoing major abdominal surgery and found that patient-controlled analgesia was associated with a reduced risk of such complications [7]. The predominance of pulmonary events in the present study may reflect the combined effects of abdominal incision, postoperative pain, reduced diaphragmatic excursion, atelectasis, immobility, and comorbid cardiopulmonary disease. These findings emphasize the importance of adequate analgesia, chest physiotherapy, early mobilization, incentive spirometry where available, and careful postoperative respiratory monitoring.

The findings of this study have practical implications for perioperative care. Several of the identified predictors, including ASA status, hypoalbuminemia, emergency surgery, and prolonged operative duration, can help clinicians identify patients who require closer surveillance. Other factors, particularly intraoperative hypotension and blood transfusion, may represent modifiable targets for perioperative optimization. A structured approach incorporating preoperative risk assessment, nutritional optimization, anemia management, careful hemodynamic control, rational transfusion practice, effective analgesia, and early postoperative mobilization may help reduce the burden of postoperative medical complications. Importantly, the purpose of this study was not to validate a formal risk prediction model, but to identify clinically relevant factors associated with postoperative medical morbidity in a contemporary multicenter abdominal surgery cohort.

Strengths and limitations

This study has several strengths. It was designed as a prospective observational cohort and included consecutive patients undergoing major abdominal surgery at two tertiary care centers. The inclusion of both elective and emergency procedures improves the clinical relevance of the findings, as this reflects real-world abdominal surgical practice. In addition, the study evaluated a broad range of preoperative and intraoperative variables and focused specifically on postoperative medical complications within 30 days, rather than limiting the outcome to technical surgical complications.

However, several limitations should be acknowledged. First, although the study was multicenter, recruitment was limited to two tertiary care hospitals in a single city, which may limit the generalizability of the findings to other healthcare settings. Second, some variables, such as delirium and systemic infection, were based on clinical documentation and treating-team assessment, which may introduce variability in diagnosis. Third, postoperative complications were assessed over a 30-day period, and longer-term outcomes, such as readmission, long-term mortality, and functional recovery, were not evaluated. Fourth, although multivariable regression was used to adjust for confounding, residual confounding remains possible, particularly for factors such as surgical complexity, severity of emergency presentation, baseline frailty, and differences in perioperative care. Finally, intraoperative transfusion and hypotension may partly reflect greater operative severity rather than fully independent causal factors, and the use of serum albumin <3.0 g/dL as the threshold for marked hypoalbuminemia may have underestimated the overall burden of hypoalbuminemia compared with the conventional <3.5 g/dL cut-off. Therefore, the findings should be interpreted as reflecting the association between postoperative complications and more marked albumin depletion rather than mild hypoalbuminemia. Although predefined operational criteria were used to identify postoperative medical complications, some outcomes still depended on the quality and completeness of clinical documentation. In particular, formal protocol-based screening for delirium using CAM, CAM-ICU, or 4AT was not routinely performed across all sites. Similarly, full Sepsis-3 scoring could not be applied uniformly in all cases because this was an observational clinical study based on available perioperative records. Therefore, outcome misclassification and differential ascertainment cannot be completely excluded.

A further limitation is the potential for surveillance or detection bias. Patients with higher preoperative risk, emergency surgery, ICU admission, or greater postoperative clinical instability may have undergone more frequent clinical assessment, laboratory testing, imaging, and specialist review. As a result, complications such as myocardial injury, infection, delirium, or pneumonia may have been more readily detected in these patients compared with lower-risk patients managed on routine wards. Although objective criteria were used where available, some outcomes depended on documentation by the treating clinical team, and protocol-based screening for delirium, sepsis, and myocardial injury was not uniformly performed across all sites. Therefore, the observed associations between perioperative risk factors and postoperative medical complications should be interpreted with this potential differential detection in mind.

## Conclusions

Postoperative medical complications remain a significant source of morbidity following major abdominal surgery, with approximately one-third of patients in this multicenter cohort developing at least one complication within 30 days of surgery. Pulmonary complications, acute kidney injury, and systemic infection were the most frequently observed events. Higher ASA physical status, hypoalbuminemia, emergency surgery, prolonged operative duration, intraoperative hypotension, and intraoperative blood transfusion were independently associated with postoperative medical complications. These findings highlight the importance of comprehensive perioperative assessment and optimization, particularly in patients with reduced physiological reserve and those undergoing complex or emergency procedures. Several identified factors, including hemodynamic instability and transfusion exposure, may represent potentially modifiable perioperative targets. Early identification of high-risk patients, combined with careful perioperative management, nutritional optimization, effective hemodynamic control, and appropriate postoperative monitoring, may help reduce postoperative medical morbidity after major abdominal surgery. Further multicenter studies with larger cohorts and longer follow-up are needed to validate these findings and explore strategies aimed at reducing postoperative medical complications in high-risk surgical populations.

## Appendices

**Table 4:** Candidate variables considered for multivariable logistic regression. EPV: events per variable; ASA: American Society of Anesthesiologists

Candidate variables	Reason for consideration	Included in final model	Reason if excluded
Age	Univariate p<0.10/clinically relevant	Yes/no	Excluded if not retained due to limited EPV or loss of independent association after adjustment
ASA ≥III	Univariate p<0.10/clinically relevant	Yes/no	-
Diabetes mellitus	Univariate p=0.028	No	Considered, but not included in the restricted model because of limited outcome events and prioritization of stronger perioperative predictors
Ischemic heart disease	Univariate p=0.031	No	Considered, but not included in the restricted model because of limited outcome events and prioritization of stronger perioperative predictors
Emergency surgery	Univariate p<0.10/clinically relevant	Yes/no	-
Open surgical approach	Univariate p<0.10/clinically relevant	Yes/no	Excluded if not retained due to limited EPV or loss of independent association after adjustment
Marked hypoalbuminemia	Univariate p<0.10/clinically relevant	Yes/no	-
Intraoperative blood transfusion	Univariate p<0.10/clinically relevant	Yes/no	-
Intraoperative hypotension	Univariate p<0.10/clinically relevant	Yes/no	-
ICU admission	Univariate OR: 3.88, p<0.001	No	Not included in the primary predictor model because ICU admission may reflect postoperative severity, treatment escalation, or early complications rather than an independent preoperative/intraoperative predictor
Poor postoperative pain control	Univariate p=0.011	No	Not included in the primary predictor model because it is a postoperative factor and may lie on the causal pathway between surgery, complications, and escalation of care
